# A ^68^Ga-/Gd labeled PET/MR imaging probe for pH assessment

**DOI:** 10.1186/s13550-025-01330-7

**Published:** 2026-01-13

**Authors:** Noémi Kovács, Imre Hegedüs, Kálmán Nagy, Eliana Gianolio, Roberta Napolitano, Francesca Arena, Bengt Långström, Krisztián Szigeti, Miklós Tóth, Balázs Gulyás, Domokos Máthé, Christer Halldin, Silvio Aime

**Affiliations:** 1 Department of Biophysics and Radiation Biology - HUN-REN TKI Neuroinflammation Research Group, Semmelweis University, Budapest, Hungary; 2 In Vivo Imaging Advanced Core Facility, Hungarian Centre of Excellence for Molecular Medicine, Budapest, Hungary; 3 Mediso Medical Imaging Systems Ltd, Budapest, Hungary; 4 Department of Molecular Biotechnology and Health Sciences, University of Turin, Turino, Italy; 5 Department of Chemistry, Uppsala University, Uppsala, Sweden; 6 Karolinska Insitute PET Centre, Stockholm, Sweden; 7 Cognitive Neuroimaging Center, Nanyang Technological University, Singapore, Singapore; 8 IRCCS SDN SynLab, Naples, Italy

**Keywords:** Dual PET/MRI functional imaging system, Parallel pH measurement and localization, Gd-DO3a-sulphonamide derivative, ^68^Ga-NOTA-chelator, Avidin/biotin system

## Abstract

**Supplementary Information:**

The online version contains supplementary material available at 10.1186/s13550-025-01330-7.

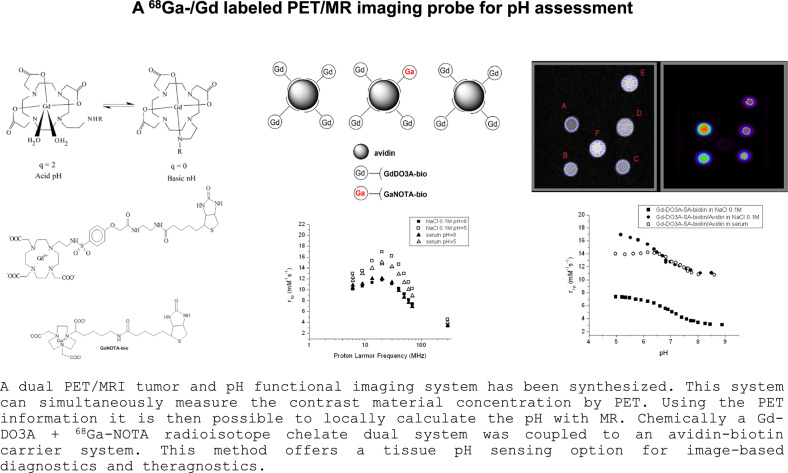

## Introduction

Currently, there is a growing interest to acquire in vivo images by integrating different modalities. Much attention is focused on the combination of MRI and PET, as they can provide complementary information [1]. The integration of the imaging modalities implies the design of novel hybrid probes that exploit the complementary properties of the involved imaging techniques [2–7]. The consequent additive information from the two techniques may be particularly effective in the assessment of the effect of medications in oncological tissue [8, 9]. Several attempts have been made to detect the change in the metabolic rate of tumors. In many cases, the effect of treatment on tumor metabolism can be detected by measuring extracellular pH changes using magnetic resonance imaging [10–13]. Lower extracellular pH values (pH = 6.8–6.9) can be measured in cancerous tissues in respect to healthy ones (pH = 7.2–7.4). This pH decrease in the extracellular space is due to increased lactic acid production in tumor cells [14, 15]. At the boundary between healthy and cancerous tissue, the pH value changes within hundreds of micrometers [16, 17], and pH measurements can be used to determine the boundary between healthy and cancerous tissue with such accuracy [17]. Lower extracellular pH anticipates a lower tumor sensitivity to external radiation therapy.

A combination of MRI and PET measurements for the detection of tumor metabolism, radiation sensitivity, and pH changes has been reported [18–21]. Both techniques provide primary information for surgeons guiding more precise resection of malignant tissues from the healthy ones. A dual imaging reporter probe, when the imaging reporter moieties are part of the same molecule, optimally reports on the same biodistribution in the respective images, thus enhancing the ability to delineate the tumor lesion. This task was nicely accomplished for the pH-responsive MRI-PET agent reported by Caravan et al. [6]. Their approach consisted of conjugating a pH-responsive Gd complex to an F-18 containing synthon. From the PET detection of F-18 reporting units, the quantitative evaluation of the concentration of the agent was obtained and used to transform the observed MRI-R_1_ values (R_1_ = 1/T_1_) into the pH-dependent relaxivities (r) map. More recently [20], another PET/MRI procedure was reported for sensing tumor extracellular pH based on the co-administration of a pair of contrast agents consisting of a pH-responsive MRI probe (Gd-DOTA-sulfonamide) and a pH-independent PET coagent (^68^Ga-DOTA-sulfonamide). These two agents were designed to have identical pharmacokinetics, due to the same residual charge and identical DOTA-derivative for chelation, so that a dual injection of the two agents at a known ratio would allow for a quantitative measure of pH. While in vitro phantom studies demonstrated the ability of the system to accurately measure pH in solution, in vivo studies in a subcutaneous tumor model of a pancreatic cancer allowed us to conclude that the PET/MRI co-agents can be used to monitor pH in the tumor microenvironment only up to 16 min after administration. This limitation was ascribed to the fast in vivo degradation of the radiolabeled complex. The choice of a DOTA derivative for ^68^Ga^3+^ complexation, even if dictated by the design of the proposed system, is in fact not the best one in terms of thermodynamic stability of the formed complex (logK_ML_ = 21.3) [22]. Moreover, DOTA is not selective for ^68^Ga^3+^ and forms equally strong complexes with many other endogenous metal ions.

In this communication, we report another approach to dual PET/MRI probes based on the formation of supramolecular adducts between avidin and biotinylated derivatives of a Gd-DOTA-sulfonamide and ^68^Ga-NOTA. As in the previously reported system, the Gd-complex acts as a pH-responsive MRI agent, whereas the ^68^Ga complex provides a way to access the exact local concentration of the agent by its PET response. Here, the co-localization of the two agents is guaranteed by their very strong binding to the same macromolecular carrier, Avidin. Biotin, in fact, owns a very high affinity (K_a_ = 10^15^) [23] towards avidin (M_w_ = 66 kDa). On the other hand, a very high thermodynamic stability of the radiolabeled complex is obtained by using NOTA ligand as a chelator, which is known to be one of the strongest chelators for ^68^Ga^3+^ (logK_ML_ = 31.0) [24]. This extraordinarily high stability constant of ^68^Ga-NOTA complex is attributed to the compatibility of the ionic size of ^68^Ga^3+^ with the cavity dimension of the nine-membered triazamacrocycle of NOTA. The positron-emitting radionuclide ^68^Ga (T_1/2_ = 68 min; 89% β^+^-emission) is available from a long shelf-life ^68^Ge/^68^Ga generator provided by a 270-day half-life of the parent ^68^Ge. The half-life of ^68^Ga permits ^68^Ga-based radiopharmaceuticals to be easily produced and used onsite.

The Gd-DOTA-sulfonamide derivatives modulate their hydration state as a consequence of the change in the denticity of the ligand that is heptadentate at acidic pH and becomes octadentate at basic pH following the deprotonation step of the sulfonamide moiety (Chart S1) with pK_a_ around the physiologically relevant values (6.5–7) [25].

## Materials and methods

All commercially available reagents used in the synthesis were obtained from Sigma Aldrich and TCI and used without further purification. All the reactions were monitored by HPLC (Column: Zorbax SB-Phenyl 4.6 × 250 mm), eluents: 0.1% formic acid and acetonitrile. Reverse-phase chromatographic purifications were performed on an automated purification system (AKTA pure), on Sephadex G10 or Amberchrom CG161 resin, generally eluting with a water-acetonitrile gradient. The purity of every compound synthesized was determined through HPLC − UV and HPLC − ESI–MS by the ratio of the integrated HPLC peak area for the compound of interest to the integrated HPLC peak area for all peaks, obtaining for the final ligands and complexes a purity of > 95%. All the syntheses are described in detail in the Supporting Information.

### Synthesis of Gd-DO3A-SA-Biot

The pH-responsive ligand DO3A-SA-Biot has been synthesized according to the route shown in Supplementary Materials, Scheme S1. The sulfonamide bond, responsible for the pH-dependent coordination, was introduced by reaction of the intermediate amino-ethyl DO3A-tris-*tert*-buthyl ester with the commercially available methyl 2-(4-(chlorosulfonyl)phenoxy) acetate in acetonitrile. After hydrolysis of the methyl ester in basic media and coupling with benzyl (2-aminoethyl) carbamate in DMF, the benzyloxycarbonyl protecting group was removed by hydrogenation with Pd/C at RT. The free amino group was then coupled to D-Biotin using TBTU/DIPEA in DMF protocol, and after removal of the *tert-*butyl ester protecting groups by treatment with trifluoroacetic acid, ligand DO3A-SA-Biot was obtained sufficiently pure by precipitation with diethyl ether. The complexation with Gd(III) has been done according to the method described in ref [26], allowing a slight excess of the ligand (typically 2 − 3%) to ensure that all Gd(III) is under the complexed form. Salts were then removed by purification on Sephadex G10 resin, ensuring the recovery of the final complex with purity > 98%.

### Synthesis of NOTA-Biot

The NOTA-Biot ligand has been synthesized according to the route (see Supplementary Materials, Scheme S2). 1,4,7-Triazacyclononane was monoalkylated with tert-butyl 6-{[(benzyloxy)carbonyl]amino}−2-bromohexanoate (prepared after bromination of N_6_-Carbobenzyloxy-L-lysine and then protection of the carboxyl group as *tert*-butyl ester) in acetonitrile. After alkylation with *tert*-butyl bromoacetate and removal of the benzyloxycarbonyl protecting group by hydrogenation with Pd/C at RT, the resulting free amino group was coupled with D-Biotin using TBTU/DIPEA in DMF protocol. The pure ligand was obtained after treatment with trifluoroacetic acid and purification on Amberchrom CG161 resin with a water–methanol gradient. The feasibility of the complexation was evaluated with cold GaNO_3_ at pH 5.8, reaching completion after 10 min at 90 °C, as confirmed by LC–MS.

### Synthesis of Ga-NOTA-Biot complex

Regarding the PET active compound, the biotin moiety has been conjugated to the NOTA scaffold through a lysine residue. The complexation with gallium was attained by heating the ligand with Ga(NO_3_)_3_ in water at 90 °C, pH 5.8, for 10 min. The complexation reaction was first checked with ^69^ Ga, and the complex formation was verified by ^1^H, ^13^C, and ^71^Ga NMR, using Ga(NO_3_)_3_ as a standard.

### Formulation of the dual ⁶⁸Ga/Gd imaging probe

In formulating the dual ⁶⁸Ga/Gd imaging probe, the significant difference in sensitivity between PET and MRI modalities must be carefully accounted for. To this end, a final molar ratio of 3.9:1 for Gd-DO3A-SA-Biot to avidin was employed, ensuring that a sufficient number of binding sites remain available for the ⁶⁸Ga-NOTA-Biot complex [30]. This strategy yields a dual probe predominantly composed of adducts containing four Gd complexes, with a minority incorporating three Gd complexes and one ⁶⁸Ga complex (see Chart S2). Both adduct types possess the same net charge and nearly identical molecular weights, allowing the reasonable assumption that they will exhibit comparable biodistribution profiles in vivo.

### Relaxation rate measurements

First, the relaxation rate (R_1_) values were measured by inversion recovery at 21 MHz (0.47 T) and 25 °C using a Stelar SpinMaster spectrometer (Stelar Snc, Meade (PV), Italy) as a function of solution pH. Temperature was controlled with a Stelar VTC-91 airflow heater, and the temperature inside the probe was checked with a calibrated RS PRO RS55-11 digital thermometer. Data were acquired using a recovery time ≥ 5xT1 and with 2 scans per data point. The absolute error in R_1_ measurements was less than 1%.

NMRD profiles were recorded on a Stelar SpinMaster Fast Field-Cycling (FFC) relaxometer at a continuum of proton frequencies from 0.01 MHz to 20 MHz; additional points were obtained between 21.5 MHz and 80 MHz with a Bruker WP80 electromagnet coupled to a Stelar SpinMaster spectrometer. Both systems were equipped with Stelar VTC-91 temperature control, and the internal temperature was checked with a calibrated RS PRO RS55-11 digital thermometer. NMRD profiles were fitted using the classical Solomon–Bloembergen–Morgan theory.

To calibrate the pH-dependent relaxivity under conditions matching those of the planned imaging experiments on an integrated PET/MRI(1 T) scanner (nanoScan PET/MRI, Mediso Ltd.), relaxivity measurements were also performed on an Aspect 1 T MRI scanner (Aspect Imaging Ltd., Rehovot, Israel). T_1_ maps of a phantom consisting of seven tubes filled with Gd-DO3A-SA-Biot/Avidin (in NaCl 0.1 M) at different values of pH in the range 5–8 were acquired and the corresponding relaxivity values reported in Figure S1. In the investigated pH range, the mutual dependence of r_1_ from pH is linear, and the corresponding interpolation line is r_1_ = −3.62 × pH + 35.39. T1 values were measured using an inversion − recovery spin − echo sequence (TE = 8 ms, 10 variable TR ranging from 40 to 4000 ms, NEX = 7, FOV = 3 × 3 cm^2^, 1 slice 2 mm).

The concentrations of Gd complex solutions, for the relaxometric characterization, were determined by a relaxometric procedure after mineralization of the sample in conc. HCl at 120 °C overnight. The procedure was as follows: gadolinium complex solutions were mixed in equal volumes with 37% HCl and heated in sealed vials at 120 °C overnight to solubilize the free Gd^3+^ aqua ion. The R_1_ of the solution was measured at 25 °C and 21.5 MHz, and the concentration was determined using the equation: R_1_ = R_1d_ + r_1p_ × [Gd]. Where R_1d_ is the diamagnetic contribution (0.5 s^−1^) and r_1p_ is the relaxivity of free Gd(III) aqua ions (13.5 mM^−1^ s^−1^) under the same experimental conditions (strongly acidic pH, 20 MHz, and 298 K).

### PET/MRI imaging

The work has been carried out on the PET/MRI scanner (Mediso nanoScan PET/MRI system) operating at 1 T magnetic field on phantom containing solutions with externally validated pH (using pH electrode) from 4.93 to 7.74. First, the Ga-NOTA-Biot/avidin adduct was prepared by dissolving the Ga complex, collected from the generator, and avidin in a buffer solution. Then, a known amount of Gd complex was added, and 5 solutions were obtained by successive dilution from the mother solution. The pH of each vial was adjusted to the values reported in Table 2 (measured pH electrode).

**Fig. 1:**
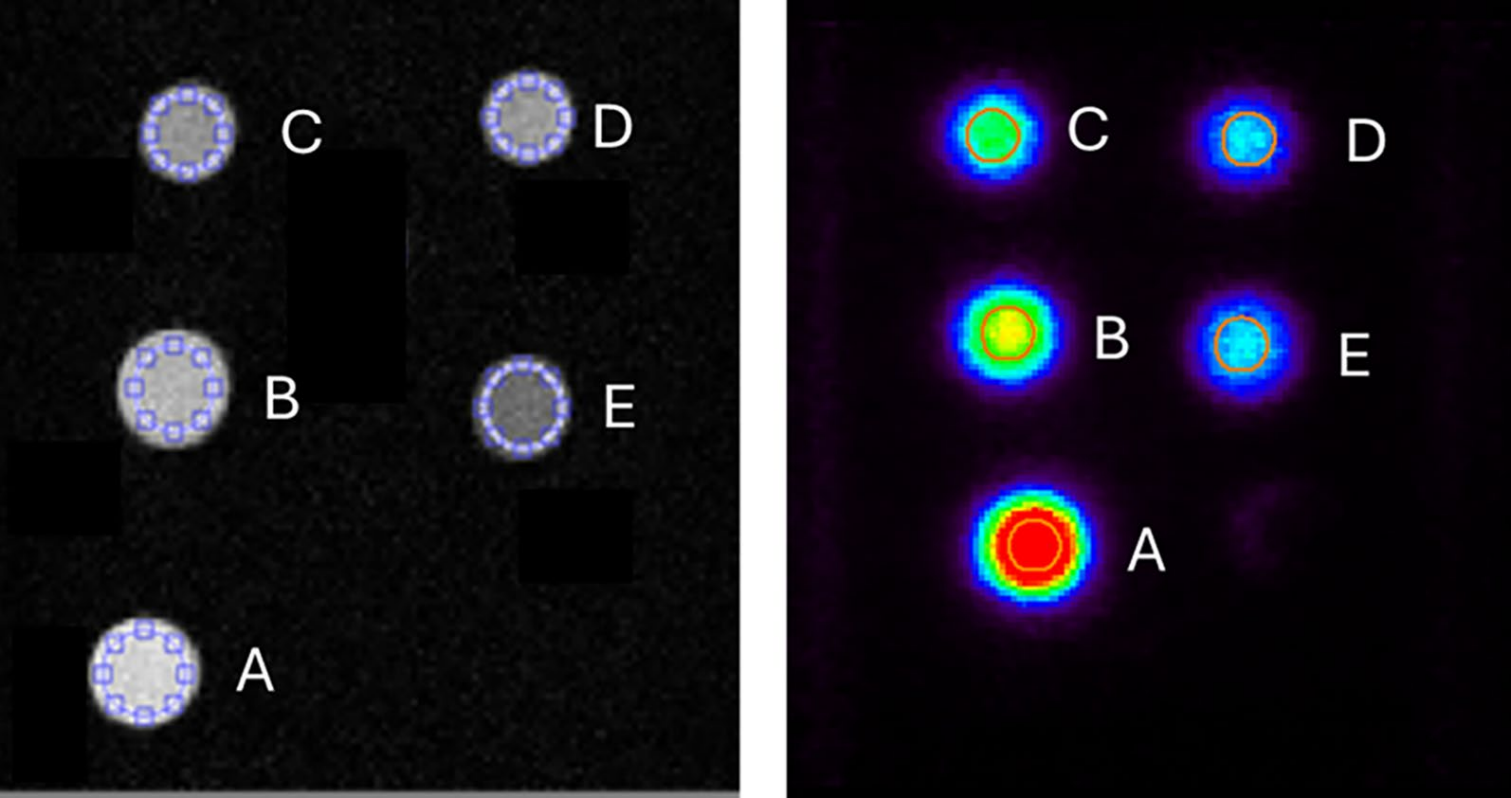
The MRI (left side) and PET scans (right side) of the dual imaging agent in five different concentrations and pH (**A-E**)

A 20 min PET acquisition and a series of inversion recovery spin echo scans (0.4 × 0.4 × 3 mm matrix, TE/TR = 5000/9 ms) were acquired with TI between 30 and 3000 ms (Fig. 1).

MR relaxation time T_1_ was evaluated by defining circular ROIs over the MR slices covering the central region of the samples and fitting the mean value in the ROIs as a function of TI with the expression $P1 \cdot \left| {1 + e^{{ - TR/T_{1} }} - 2 \cdot e^{{ - TI/T_{1} }} } \right|$. List mode PET data were binned using a 400–600 keV energy window, a 5 ns time window. Activity concentrations from 3.2 to 12.6 MBq/mL were imaged in PET and reconstructed with a three-dimensional maximum a posteriori-expectation maximization (3D MAP-EM, Tera-Tomo, Mediso, Hungary) algorithm. 3D isocontour VOIs were used to read the activity values.

### MRI investigation of the in vivo biodistribution and tumor uptake

The tumor model employed in this study entailed the subcutaneous inoculation of B16/F10 mouse melanoma cell line into three female BALB/C mice. All mice were maintained under environmentally controlled conditions, including 12-h light/dark cycles, a temperature range of 20–23 °C, and a relative humidity of 50%. They had access to food and water ad libitum. The mice were group-housed in well-ventilated cages with appropriate cage enrichment. Each mouse was assigned an ear tag for identification purposes, and randomization was applied to mitigate measurement bias. All procedures involving the animals adhered to national and international regulations concerning experimental animals (L.D. 26/2014; Directive 2010/63/EU) and received approval from the Committee on Animal Care.

MR images were obtained 10–13 days after the inoculation of the tumor, with tumor dimensions ranging from 80 to 200 mm^3^. To administer the contrast agent, an intravenous catheter was inserted into the tail vein of the animal while it was under anesthesia, prior to positioning it inside the MR scanner.

The animals were anesthetized by intramuscular injection of a combination of Tiletamine/Zolazepam (Zoletil 100, Virbac, Milan, Italy) at a dosage of 20 mmol kg^−1^ and xylazine (Rompun; Bayer, Milan, Italy) at a dosage of 5 mmol kg^−1^. To maintain the body temperature of the mouse, a heated pad was used in addition to monitoring the breathing rate using an air pillow placed beneath the animal (SA Instruments, Stony Brook, NY, USA).

Mice were injected with either 0.05 mmol Gd/kg of either Gd-DO3A-SA-Biot or the Gd-DO3A-SA-Biot/Avidin supramolecular complex (3:1 molar ratio). MR images were acquired at 7 T using a Bruker Pharmascan MRI system equipped with a 35 mm 1H/1H volume coil.

A series of T1-weighted MSME scans were acquired before and after (up to 6 h) the intravenous administration of the gadolinium complexes to track the kinetics of the contrast agent in the tumor, kidneys, bladder, liver, and tumor. The scans were performed with the following parameters: TR = 250 ms, TE = 8.9 ms, number of averages = 5, FOV = 45 mm × 45 mm, slice thickness = 1.5 mm, matrix size 128 × 128, spatial resolution = 0.375 mm per pixel × 0.375 mm per pixel, acquisition time = 2 min and 40 s. A 5 mm NMR glass tube containing 0.5 mM of ProHance in water was inserted near the mouse body as a reference.

After acquiring the images, the T_1_ contrast enhancement (SE%) was calculated using the following formula: SE% = [(SI)post—(SI)pre]/(SI)pre × 100, where (SI)post and (SI)pre represent the signal intensities (normalized by dividing for the external standard reference) after and before the injection of both Gd(III)-contrast agents, respectively. Regions of Interest (ROIs) were manually delineated within the reference standard sample, tumors, kidneys, bladder, and various regions of the liver. The SE% was calculated in all the ROIs as described above.

## Results and discussion

**Fig. 2:**
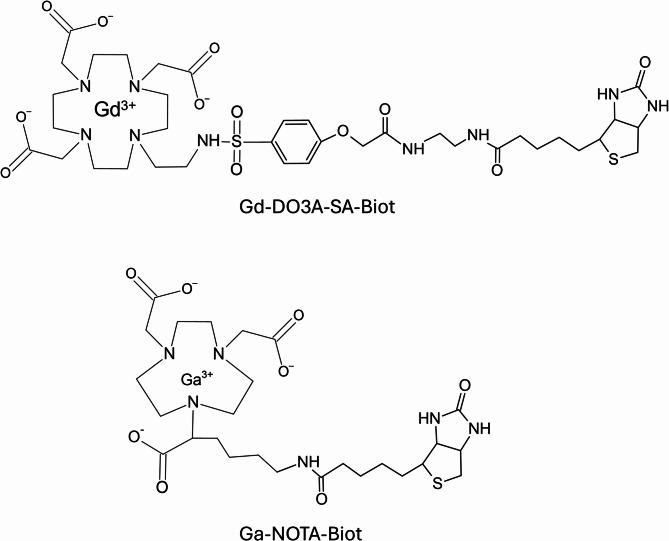
Chemical structures of the biotinylated Gd(III)-DO3A-sulfonamide and Ga-NOTA complexes

The Gd(III)-DO3A-sulfonamide complex was conjugated to biotin (Gd-DO3A-SA-Biot), and an analogous procedure has been applied to generate the corresponding biotinylated ^68^ Ga complex (Ga-NOTA-Biot) (Fig. 2).

**Fig. 3:**
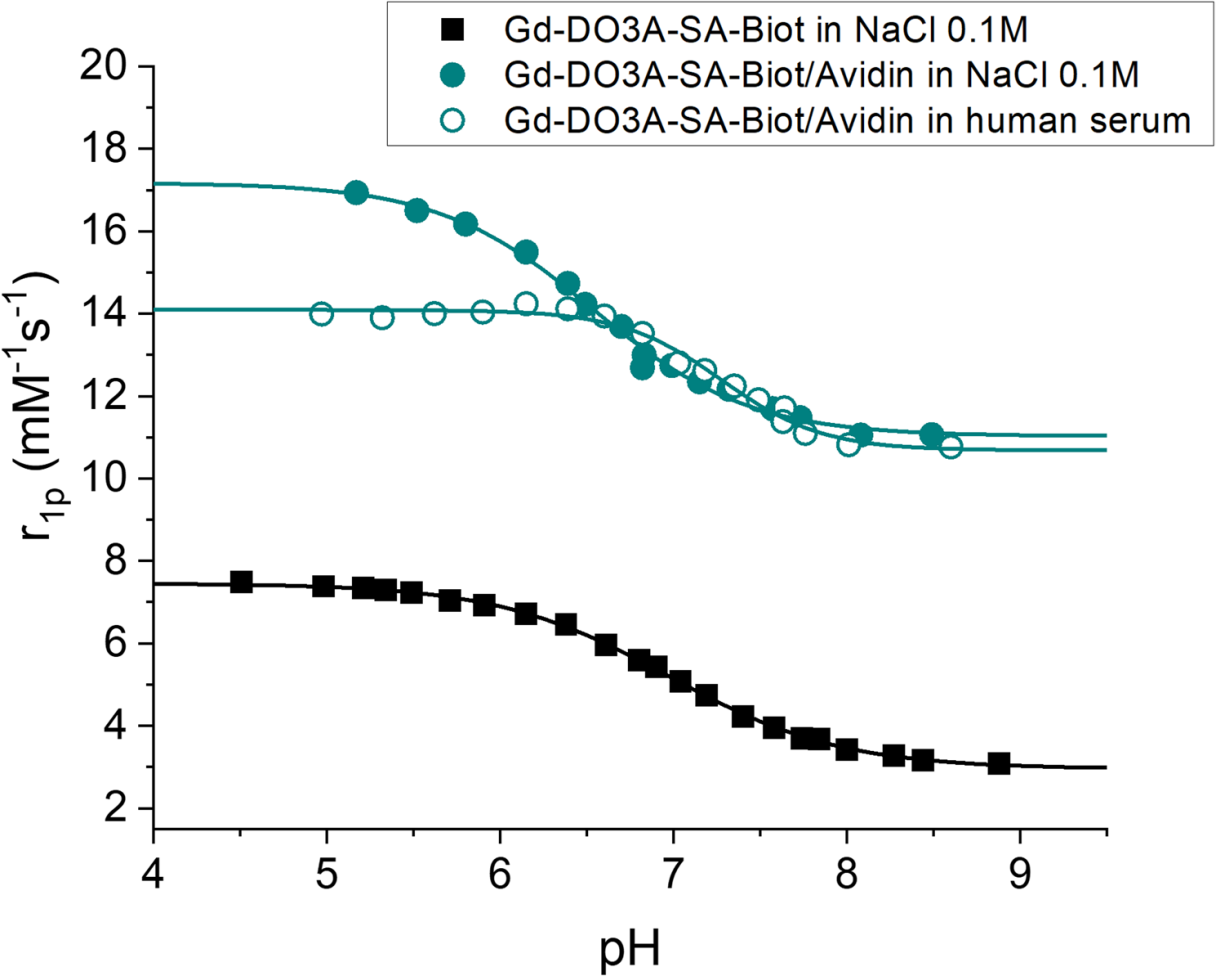
The pH dependency of the relaxivity values of Gd-DO3A-SA-Biot measured in NaCl 0.1 M (∎) and of its adduct with Avidin measured in NaCl 0.1 M (●) or in human serum (○) at 21 MHz and 298 K

First, it has been confirmed that the introduction of the biotin moiety on the DO3A sulfonamide does not modify the pH dependence of the previously reported longitudinal relaxivity of the parent compound [25]. The pH dependence of the change in relaxivity (at 21 MHz and 298 K) of the free Gd-DO3A-SA-Biot complex in saline solution revealed a sigmoidal curve (Fig. 3) characteristic of a simple two-state equilibrium process. Analysis by iterative least-squares fitting with a Boltzmann equation afforded an apparent protonation constant of the sulfonamide group (pK_a_) of 6.97 ± 0.01. At low pH, the limiting relaxivity was 7.4 mM^−1^ s^−1^ and fell to a value of 2.95 mM^−1^ s^−1^ at basic pH. This behavior mirrors the mode of action of other previously reported Gd-DO3A-sulfonamide derivatives [4, 20, 27, 28] and is based on the change in inner sphere hydration. Upon deprotonation, the sulfonamide, bearing a negatively charged nitrogen, enters into the coordination sphere of the Gd(III) ion, replacing the two water molecules and causing a marked drop of relaxivity.

**Fig. 4:**
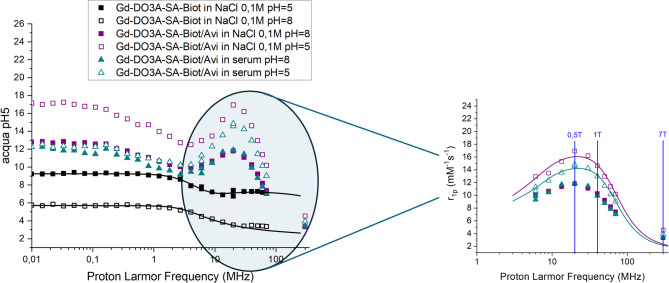
The NMRD profiles of the supramolecular adduct Gd-DO3A-SA-Biot/Avidin measured at pH 5 and 8 in NaCl 0.1 M solution or in human serum

Upon the formation of the supramolecular adduct (molar ratio Gd: avidin was 3.9:1), the lengthening of the molecular reorientational time (τ_R_) yields an increase in relaxivity, particularly pronounced at pH lower than 6 (Fig. 3). In fact, the relaxivity of the adduct at acidic pH tends to a value of 17.2 mM^−1^ s^−1^ (298 K, NaCl 0.1 M, 21 MHz) while in basic conditions is reduced to 11.0 mM^−1^ s^−1^. This gain in relaxivity at the field of ca. 0.5 T is evidenced by the NMRD profiles measured at pH 5 and 8 (Fig. 4) and is typical of large adducts. Moving to higher fields (i.e., 7 T, 300 MHz proton frequency), the pH dependence of relaxivity of the system is almost completely lost. When the relaxivity of Gd-DO3A-SA-Biot/Avidin adduct was tested in human serum (Figs. 3 and 4), the pH-dependence of the relaxivity and the NMRD profiles were found to be similar to the ones measured for the adduct in saline solution. Apparent protonation constants (pK_a_) calculated from the fitting of experimental data were 6.53 ± 0.04 and 7.28 ± 0.04 in saline and in human serum, respectively. This result indicates that the Gd complex, once bound to avidin, does not interact with other serum proteins, *i.e.*, albumin, maintaining the coordination-decoordination of the sulfonamide arm on the metal ion. The smaller values of the observed relaxivity in serum at pH < 6 are ascribable to competitive interactions of endogenous ligands (such as carbonate, phosphate, carboxylate-containing systems, etc.) towards the Gd-ion that yield an overall, although limited, reduction of the metal ion hydration [29].

Fitting of the NMRD profiles (Fig. 4) using the classical Solomon–Bloembergen–Morgan theory was performed for Gd-DO3A-SA-Biot in saline at pH 5 and pH 8, as well as for the Gd-DO3A-SA-Biot/Avidin adduct in both saline and human serum at pH 5. The fit parametric results are summarized in Table 1. For the free complex, the inner-sphere hydration number (q) was reduced from 2 to 0.3 passing from acidic to basic conditions, while electronic and reorientational correlation times remained largely unchanged. In contrast, formation of the supramolecular adduct with avidin led to an approximately 200-fold increase in reorientational correlation time, both in saline and in serum, consistent with the expected slower tumbling of the larger complex. The inner-sphere contribution for the Gd-DO3A-SA-Biot/Avidin adduct measured in human serum at acidic pH was slightly lower (q = 1.75) compared to that in saline (q = 2), likely due to mild competitive binding from serum components, as discussed above.

**Table 1:** Relevant parameters obtained from fitting of NMRD profiles

	Δ^2^ (s^−2^)	τ_V_ (ps)	τ_R_ (ps)	τ_M_ (μs)	q
Gd-DO3A-SA-Biot saline, pH = 5	5.05 ± 0.7 × 10^19^	18 ± 1.2	150 ± 20	3.6 ± 0.41	2
Gd-DO3A-SA-Biot saline, pH = 8	2.14 ± 0.5 × 10^19^	32 ± 0.7	120 ± 30	0.07 ± 0.02	0.3
Gd-DO3A-SA-Biot/Avi saline, pH = 5	4.10 ± 0.5 × 10^19^	103 ± 70	27,000 ± 8000	2.5 ± 0.12	2
Gd-DO3A-SA-Biot/Avi human serum, pH = 5	4.00 ± 0.3 × 10^19^	89 ± 18	32,000 ± 9000	2.5 ± 0.35	1.75

To calibrate the pH-dependent relaxivity under conditions matching those of the planned imaging experiments on an integrated PET/MRI (1 T) scanner (nanoScan PET/MRI, Mediso Ltd.), relaxivity measurements were also performed on an Aspect 1 T MRI scanner. In the investigated pH range, the mutual dependence of r_1_ from pH at 1 T magnetic field is linear, and the corresponding interpolation line is r_1_ = −3.62 × pH + 35.39.

Then, the dual probe has been tested on an integrated PET/MRI scanner operating at 1 T magnetic field on a phantom containing five solutions of the dual imaging probe with validated pH (using pH electrode). The solutions were characterized by having different pH and different concentrations of Gd and ^68^ Ga, but, as they were obtained from the same mother solution, they all have the same ^68^ Ga/Gd ratio.

The PET/MRI images of the phantom are reported in Fig. 1.

**Fig. 5:**
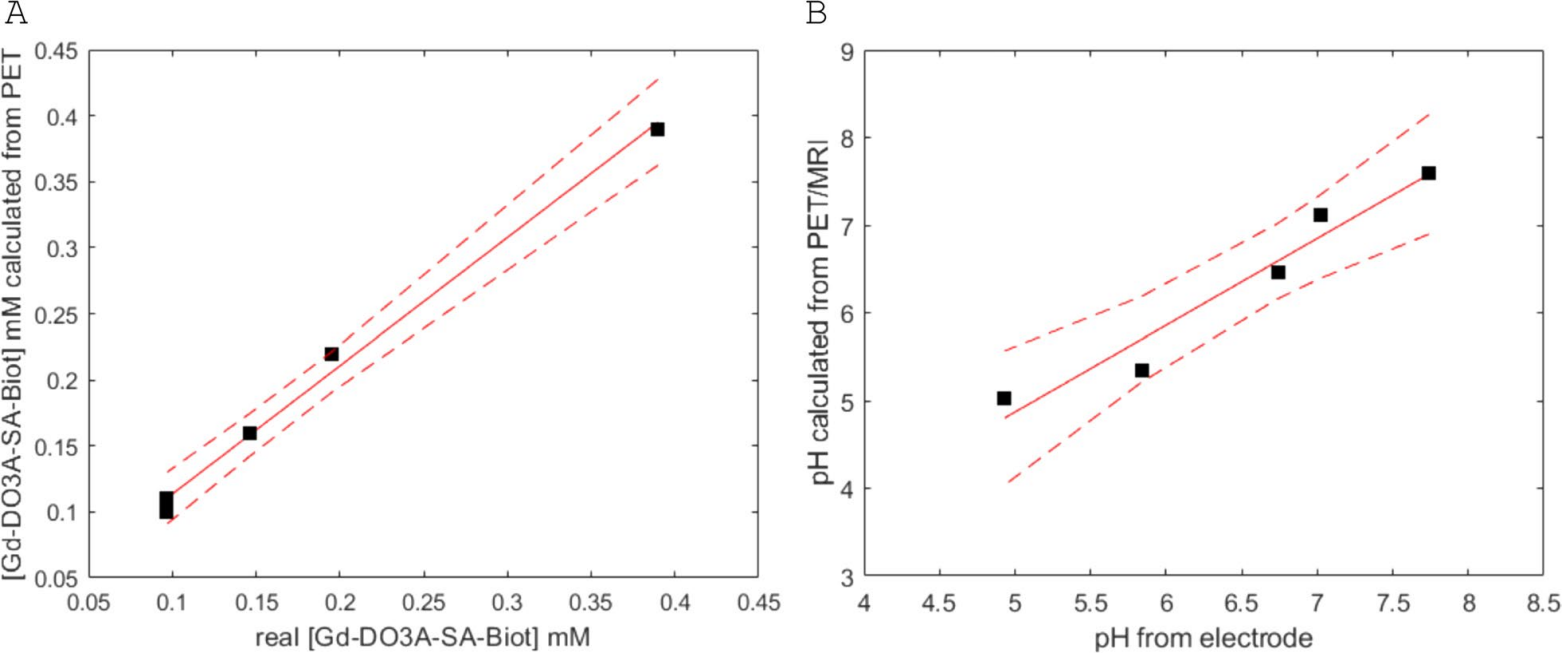
**A** Linear correlation between the Gd-DO3A-SA-Biot concentration calculated from PET measurements and its real concentration independently measured using a relaxometric method; **B** Linear correlation between the pH calculated through the PET/MRI method and that measured by a calibrated electrode

The MR T1 relaxation times in the 5 vials are determined both by the concentration of the Gd(III) complex and the pH values characterizing each solution. Conversely, in the PET image, the intensity is directly proportional to the ^68^ Ga-complex concentration. Therefore, knowing that the initial radioactivity of the mother solution is associated with a defined Gd concentration and taking into account the correction for the time decay, it was possible to calculate the Gd concentration in each phantom (Table 2). The Gd concentrations calculated in this way closely match the actual real concentrations of the analyzed samples (independently measured using a relaxometric method), as evidenced by the slope of the fitted line (0.972 ± 0.045), which is very close to 1, and the strong linear relationship (R^2^ = 0.994 and Pearson’s r = 0.997) illustrated in Fig. 5A.

**Table 2:** The measured and calculated parameters of the dual imaging agent in five different concentrations (A-E) and the reference solution (F)

Vial	Real [Gd]^a^, mM	Measured pH (electrode)	Measured T_1_ relax., ms	Measured activity concentration, MBq/ml	Calculated [Gd]^b^, mM	Calculated relaxivity^c^, mM^−1^ s^−1^	Calculated pH^d^ (MRI/PET)
A	0.39	7.02	235	12.6 ± 1	0.39 ± 0.03	9.6 ± 0.8	7.12
B	0.195	6.74	312	7.2 ± 0.44	0.22 ± 0.01	12.0 ± 0.7	6.46
C	0.146	5.84	334	5.0 ± 0.45	0.16 ± 0.01	16.0 ± 1.4	5.35
D	0.097	4.93	420	3.5 ± 0.29	0.11 ± 0.01	17.2 ± 1.4	5.02
E	0.097	7.74	857	3.2 ± 0.24	0.10 ± 0.01	7.9 ± 0.8	7.59

^a^ Independently calculated by a relaxometric method

^b^ Calculated according to the following equation: [Gd] = [GdL]/(K_st_ [L]), where [GdL] and [L] is the concentration of Gd-ligand complex and ligand, respectively, in mM, and K_st_ is a thermodynamic stability constant

^c^ Calculated according to the following equation: r_1_ = (1/T_1_−0.38)/[Gd]

^d^ Calculated according to the following equation: pH = (r_1_−35.39)/−3.62

The observed T_1_ relaxations of each vial were then converted into the respective millimolar relaxivities. Next, through the use of the linear relationship between relaxivity and pH (Fig. S1), the MRI data could be used to estimate the pH. Table 2 and Fig. 5B show a good correspondence between the actual pH values measured with an electrode and those determined by PET/MRI. Although the agreement remains strong (slope of the fitted line is 0.989 ± 0.133), the linear correlation (R^2^ = 0.949 and Pearson’s r = 0.974) is slightly lower in this case but remains within an applicable measurement tolerance limit (Fig. 5B).

**Fig. 6:**
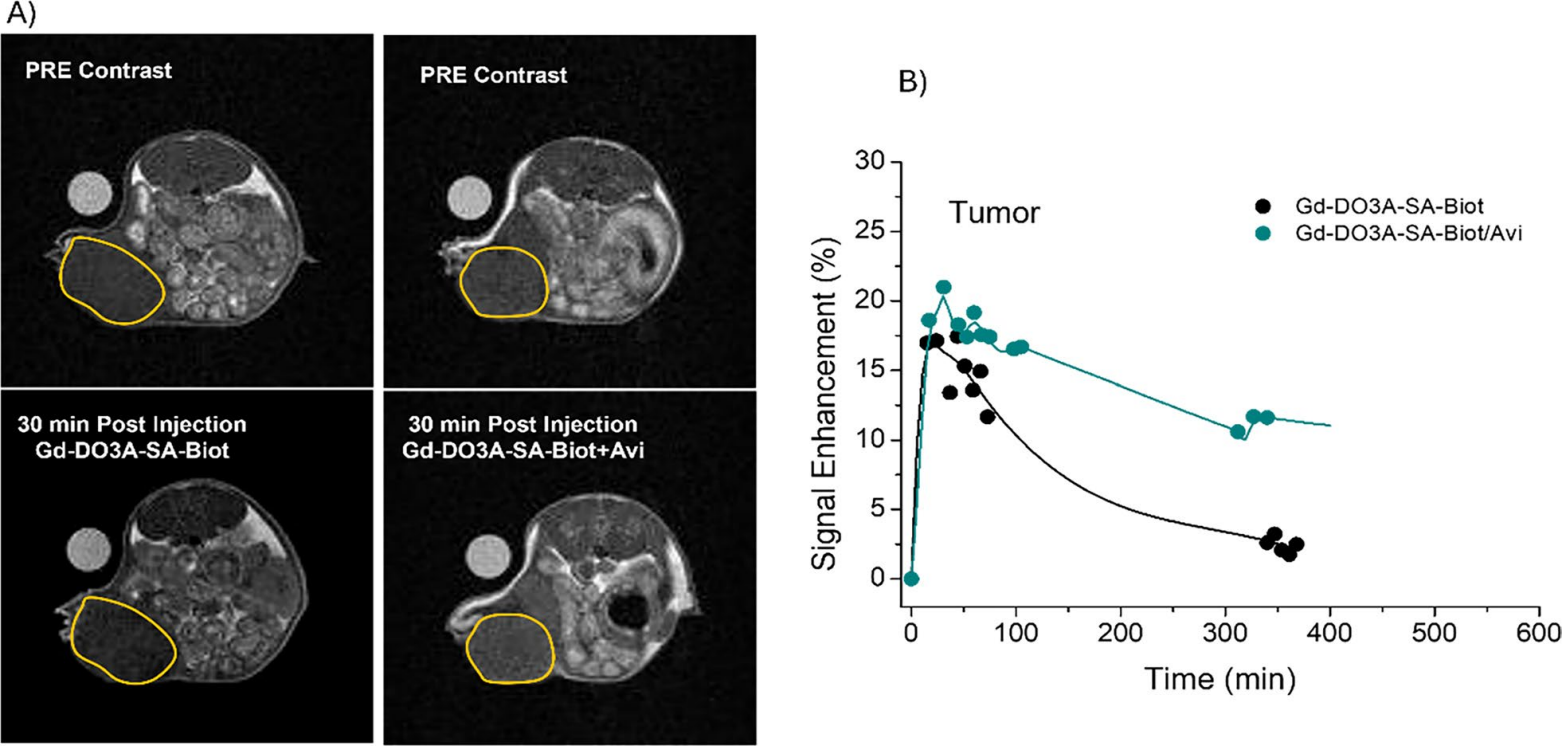
**A** T_1_ weighed MRI of mice tumor models pre and 30 min post injection of 0.05 mmol/Kg of Gd-DO3A-SA-Biot or Gd-DO3A-Biot/Avidin adduct (3:1 molar ratio). The tumor region is highlighted with a yellow circle. **B** Time dependence of the signal enhancements, with respect to pre injection, in the tumor regions of mice injected with Gd-DO3A-SA-Biot or Gd-DO3A-SA-Biot/Avidin. The solid lines are simple interpolation lines

Finally, to evaluate its potential for in vivo application, the biodistribution of the Gd-DO3A-SA-Biot probe and of its adduct with Avidin was assessed by MRI in a murine tumor model (Fig. 6A). The in vivo biodistribution of the MRI contrast agent was evaluated at 7 T, as also this scanner was available in our laboratory and the magnetic field strength does not affect the biodistribution-dependent MRI signal.

The time-dependent signal enhancement in the tumor, kidneys, bladder, and liver following intravenous injection of 0.05 mmol/kg of either Gd-DO3A-SA-Biot or the Gd-DO3A-SA-Biot/Avidin supramolecular complex (3:1 molar ratio) is shown in Fig. 6B and Figure S2.

When administered as the free complex, Gd-DO3A-SA-Biot exhibits the typical pharmacokinetic profile of small-molecule contrast agents, being rapidly filtered by the kidneys and excreted via the bladder. Complexation with avidin modestly slows renal clearance and introduces an additional hepatobiliary elimination pathway, as indicated by the marked signal enhancement observed in the liver.

Analysis of the signal enhancement in the tumor region (Fig. 6B) indicates that the supramolecular Gd-DO3A-SA-Biot/Avidin complex accumulates in tumors to a greater extent than the free Gd-DO3A-SA-Biot complex. This enhanced accumulation is likely attributable to the increased Enhanced Permeation and Retention (EPR) effect commonly associated with macromolecular agents. These findings are particularly relevant for the potential clinical translation of this probe for in vivo tumor imaging using hybrid MRI/PET scanners.

## Conclusions

The assessment of the extracellular pH in tumors and other pathologies is of paramount importance, as it is recognized as a hallmark of cellular metabolism. This task is under intense scrutiny to identify the most reliable solution for clinical translation. Images reporting on pH could also be of great interest in the monitoring of the therapeutic efficacy of the undertaken treatments. Among the several reported MRI pH-responsive systems, the most efficient ones are those based on changes of their relaxivity, often represented by Gd-containing complexes. The main limitation is related to the need to relate the observed relaxation enhancement to their actual concentration to represent the MR images in terms of the relaxivity values. The most promising approach to tackle this task appears to be the one provided by access to PET/MRI hybrid scanners. The herein reported dual agent appears to be an efficient tool to map pH in the regions where it distributes. 

In conclusion, we have described a novel MRI-PET agent that can quantitatively report on local pH. One may further speculate that the avidin platform can also provide the route to add targeting capabilities to the dual probe by using one of the four binding sites on the protein for hosting a biotinylated vector. In this way, the assessment of pH may be further localized to the anatomical region defined by the targeted sites. Moreover, the “smart” use of the multimodal avidin binding sites may suggest further applications towards the development of multi-task molecular imaging probes. In principle the herein reported method works at any magnetic field strength of the MRI scanner although the highest sensitivity is expected to occur at magnetic fields around 1 T as suggested by the acquisition of the NMRD profile on a Field Cycling relaxometer.

## Supplementary Information

Supplementary Material 1

## Data Availability

The data supporting the findings of this study can be obtained from the corresponding author upon reasonable request.

## Abbreviations

DO3A: 1,4,7,10-Tetraazacyclododecane-1,4,7-triacetic acid trisodium salt
DOTA: 2,2′,2′′,2′′′-(1,4,7,10-Tetraazacyclododecane-1,4,7,10-tetrayl)tetraacetic acid
DMF: Dimethyl formamide
DTPA: *N*,*N*′-{[(Carboxymethyl)azanediyl]di(ethane-2,1-diyl)}cbis[*N*-(carboxymethyl)glycine]
EPR: Enhanced permeation and retention
FOV: Field of view
HPLC − UV: High-performance liquid chromatography-ultraviolet
ESI–MS: Electrospray ionisation mass spectrometry
LC–MS: Liquid chromatography-mass spectrometry
MAP EM: A PET reconstruction method using the wavelet-based maximum a posteriori (MAP) expectation–maximization (EM) algorithm
NMR: Nuclear magnetic resonance
NMRD: Nuclear magnetic relaxation dispersion
MSME: Multi-spin-multi-echo pulse sequence
NEX: Number of excitations
NOTA: 2,2',2''-(1,4,7-Triazonane-1,4,7-triyl)triacetic acid (bifunctional chelator)
NOTP: 1,4,7-Triazacyclononane-1,4,7-tri(methylene phosphonic acid)
3D OSEM: 3D ordered-subset expectation maximization image reconstruction
ROI: Region of interest
RT: Room temperature
SA: Sulfonamide
TBTU/DIPEA: [Benzotriazol-1-yloxy(dimethylamino)methylidene]-dimethylazanium;*N*-ethyl-*N*-propan-2-ylpropan-2-amine;tetrafluoroborate
Tera-Tomo: 3D iterative reconstruction based on real-time monte carlo simulation ensures homogeneous image quality over the entire field of view
TE/TR: Echo time/repetition time
VOI: Volume of interest
