# Supplementary material for: A 68Ga-/Gd labeled PET/MR imaging probe for pH assessment
Source: EJNMMI Res. 2026 Jan 13;16:25. doi: 10.1186/s13550-025-01330-7 (PMC12886664; doi:10.1186/s13550-025-01330-7)
Supplement: Supplementary file 1 — Supplementary Material 1 [file 13550_2025_1330_MOESM1_ESM.docx]

**Supporting Information**

**A ^68^Ga-/Gd labeled PET/MR imaging probe for pH assessment**

**Synthesis of Gd-DO3A-SA-Biot**

**Scheme S1:** Synthesis of Gd-DO3A-SA-Biot

**Compound 2**

To a solution of Compound 1 (13.4 g, 0.023 mol, prepared as described by S. Aime et al., *J. Med. Chem.* 2010, 53, 4877-4890) in acetonitrile (75 mL), TEA (4 mL, 0.029 mol) was added and then a solution of methyl 2-(4-(chlorosulfonyl)phenoxy)acetate (6.87 g, 0.026 mol) in acetonitrile (25 mL) was slowly dropped. The solution was stirred at RT overnight, observing the formation of a white, thin precipitate that was filtered off. The solution was concentrated under reduced pressure, the oily residue was dissolved in DCM, and the organic phase was washed with water (3 x ), brine (1 x), and dried over Na_2_SO_4_. Solvent was removed under reduced pressure, obtaining a brown oil (20 g) that was used in the following step without further purification. [M+H]^+^: 786.9.

**Compound 3**

A solution of Compound 2 (20 g, 0.025 mol) in methanol (150 mL) and water (150 mL) was brought at pH 12 with 2 N NaOH and the solution was stirred at RT for 3 hours, maintaining the pH at 12 by continuous addition of 2 N NaOH (total volume added: 17.11 mL). Methanol was concentrated under reduced pressure, the resulting oil was dissolved in DCM, and the desired product was extracted with 1 N HCl (3 x). The combined aqueous phases were then neutralized with 30% NaOH, and the desired product was extracted with DCM (3 x), obtaining, after washing with brine and drying over Na_2_SO_4_, 17.8 g of a yellow oil. [M+H]^+^: 772.8.

**Compound 4**

To a solution of Compound 3 (1.14 g, 1.48 mmol) in DMF (10 mL), benzyl (2-aminoethyl)carbamate (0.29 g, 1.48 mmol), DIPEA (300 µL, 1.78 mmol), and HBTU (0.675 g, 1.78 mmol) were added, and the clear yellow solution was stirred at RT overnight. The desired product was precipitated by the addition of water (50 mL) and then dissolved in DCM (40 mL). The organic phase was washed with water (1 x), brine (1 x), and dried over Na_2_SO_4_, obtaining 1.45 g of a white oily solid. [M+H]^+^: 952.8.

**Compound 5**

Pd/C (5% Pd, 50% w/w water, 300 mg) was added under N_2_ atmosphere to a solution of Compound 4 (1.45 g, 1.52 mmol) in methanol (40 mL). N_2_ was then replaced by H_2,_ and the mixture was stirred at RT for 4 hours. The catalyst was filtered off, and the clear solution was concentrated under reduced pressure, obtaining 1.06 g of an oily solid. [M+H]^+^: 814.8

**DO3A-SA-Biot**

To a solution of Compound 5 (248 mg, 0.30 mmol) in DMF (5 mL), D-Biotin (75 mg, 0.30 mmol), DIPEA (60 µL, 0.36 mmol), and HATU (140 mg, 0.36 mmol) were added, and the resulting yellow solution was stirred at RT overnight. DMF was concentrated under reduced pressure, the oily residue was dissolved in DCM, and washed with water (3 x). The organic solution was concentrated under reduced pressure, and the resulting yellow oil (crude Compound 6) was dissolved in neat TFA (4 mL) and stirred at RT overnight. Sufficiently pure DO3A-SA-Biot was precipitated by the addition of diethyl ether (30 mL), recovering 240 mg of a white solid. [M+H]^+^: 872.6.

**Gd-DO3A-SA-Biot**

GdCl_3_ 6H_2_O (51 mg, 0.138 mmol) was added to a solution of DO3A-SA-Biot (200 mg, 60% titer, 0.138 mmol) in water kept at pH 7 by small addition of 0.1 N NaOH. pH was then increased up to 9, and the solution was stirred at RT for 4 hours, observing the formation of a thin white precipitate that was filtered off. Salts were then removed by purification on Sephadex G10, obtaining, after freeze-drying, 134 mg of a white solid. [M+Na]^+^: 1049.55.

**Synthesis of Ga-NOTA-Biot**

**Scheme S2:** Synthesis of Ga-NOTA-Biot

**Compound 7**

H-Lys(Z)-OH (21 g, 0.0749 mol) was dissolved in 47% HBr solution (60 mL), and the solution was cooled to 0 °C. Sodium nitrite (6.2 g, 0.09 mol) was added in portions over 1 hour, observing the formation of a yellow amorphous precipitate. The suspension was stirred at 0 °C for 1 hour and at RT for 2 hours, then another sodium nitrite (2.3 g) was added. After 3 hours at RT, urea (8 g) was added, and the desired product was extracted with ethyl acetate (3 x). The combined organic phases were washed with water (1 x), brine (1 x), and dried over Na_2_SO_4_, obtaining after concentration under reduced pressure Compound 7 as an orange oil (16 g), which was used in the following step without further purification.

**Compound 8**

Crude Compound 7 was dissolved in *t*-butyl acetate (250 mL), and 70% perchloric acid (1.7 mL) was dropped at RT: the orange turbid solution became immediately clear and colourless. After 4 hours of stirring at RT, 5% NaHCO_3_ was added up to pH 6, and the desired product was extracted with diethyl ether. The organic phase was then washed with water (1 x), brine (1 x), and dried over Na_2_SO_4_, obtaining a purple oil (20.7 g) that was purified on a silica column with a gradient of dichloromethane and methanol (from 0 to 10% MeOH). Fractions containing the desired product (Rf: 0.3 in 20:1 DCM:MeOH, eluted with 10:1 DCM:MeOH), were combined, obtaining Compound 8 (7.0 g) as an orange oil (titer on Br^-^: 96.6%).

**Compound 9**

A Solution of Compound 8 (1.29 g, 3.22 mmol) in acetonitrile (8 mL) was dropped at RT to a solution of 1.4.7-triazacyclononane (0.55 g, 4.2 mmol) in acetonitrile (17 mL). The solution was stirred at RT overnight. Then, acetonitrile was removed under reduced pressure, the crude oil was dissolved in dichloromethane, and washed with 0.1 M HCl (3 x). The combined aqueous phases were neutralized with 30% NaOH up to pH 8, and the desired product was extracted with dichloromethane (3 x), obtaining, after concentration under reduced pressure, pure Compound 9 as a yellow oil (620 mg), which was used in the following step without further purification. [M+H]^+^: 449.4.

**Compound 10**

*t*-Butyl bromoacetate (0.4 mL, 2.68 mmol) was added to a mixture of Compound 9 (0.60 g, 1.34 mmol) and K_2_CO_3_ (0.74 g, 5.36 mmol) in acetonitrile (20 mL). The mixture was stirred at RT for 48 hours, then the salts were filtered off and the solvent was removed under reduced pressure. The crude oil was purified on a silica column with a gradient of dichloromethane : methanol (from 7:1 to 100% MeOH). Fractions containing the desired product (eluted with MeOH) were combined and concentrated under reduced pressure, obtaining pure Compound 10 as a yellow oil (641 mg). [M+H]^+^: 677.3

**Compound 11**

Pd/C (5% Pd, 50% w/w water, 150 mg) was added under N_2_ atmosphere to a solution of Compound 10 (640 mg, 9.47 mmol) in methanol (20 mL). N_2_ was then replaced by H_2,_ and the mixture was stirred at RT for 6 hours. The catalyst was filtered off, and the clear solution was concentrated under reduced pressure, obtaining 480 mg of an oily solid. [M+H]^+^: 543.6.

**Compound 12**

D-Biotin (0.24 g, 0.097 mmol), DIPEA (150 µL, 1.06 mmol), and HATU (0.40 g, 1.06 mmol) were added to a solution of Compound 11 (0.48 g, 0.89 mmol) in DMF (10 mL), and the yellow solution was stirred at RT overnight. Water (30 mL) was added, the precipitate was filtered, dissolved in dichloromethane (30 mL) and the organic phase was washed with water (3 x), 5% NaHCO_3_ (1 x), water (1 x), brine (1 x) and dried over Na_2_SO_4_, obtaining after concentration under reduced pressure an orange oil (455 mg). The compound was used in the following step without further purification. [M+H]^+^: 769.5

**NOTA-Biot**

Compound 12 (480 mg, 0.625 mmol) was dissolved in TFA (4 mL), and the solution was stirred at RT overnight. Diethyl ether (30 mL) was added, and the white precipitate was filtered, washed with diethyl ether, dissolved in water, and purified on Amberchrom CG161M resin with a gradient of water–methanol (2 CV water, from 0 to 100% methanol in 10 CV). Fractions containing the desired pure product (eluted with 51% methanol) were combined, concentrated under reduced pressure, and freeze-dried, obtaining a white solid (269 mg). [M+H]^+^: 601.5.

**Cold Ga-NOTA-Biot**

NOTA-Biot (50 mg, 0.083 mmol) was dissolved in milliQ water (3 mL), and GaNO_3_ (21 mg, 0.083 mmol) was added. pH was adjusted to 5.8, and the solution was heated at 90 °C for 10 minutes. The solution was then cooled at RT, and salts were removed by a quick filtration on a C18 SPE, washing with water, and then collecting the desired complex with 1:1 water : acetonitrile. Solvents were removed under reduced pressure, obtaining Ga-NOTA-Biot (45 mg) as a white solid. [M+H]^+^: 668.3.


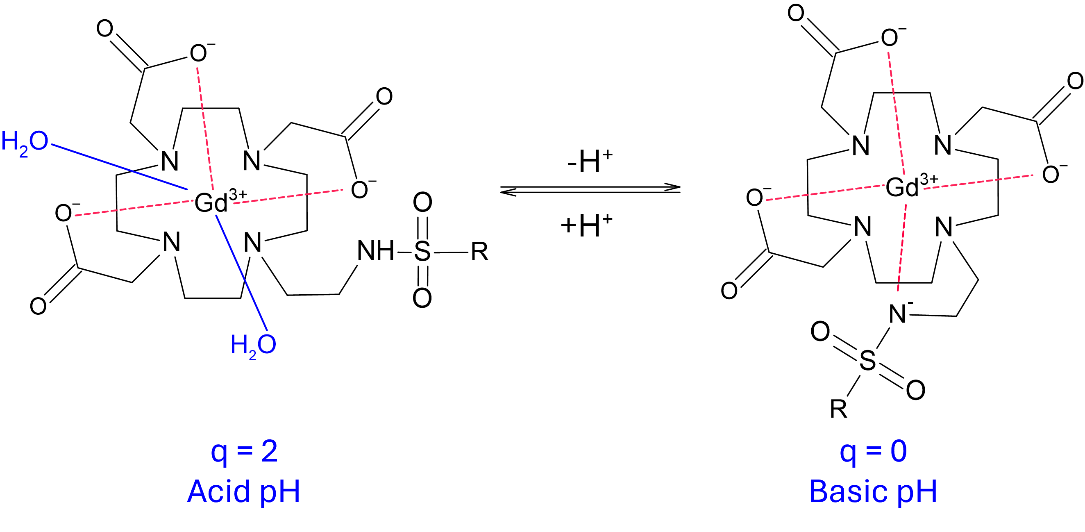


**Chart S1**: Scheme of the pH-responsive Gd-DO3A sulfonamide derivatives coordination chemistry and hydration.

**
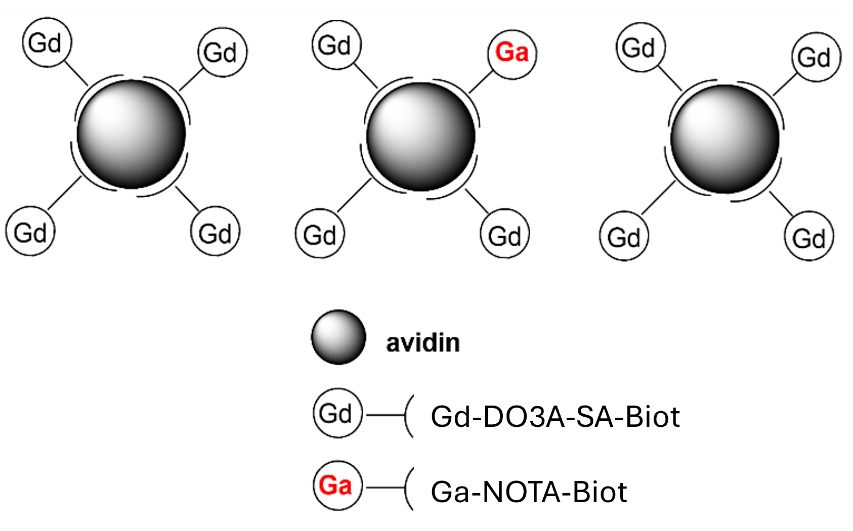
**

**Chart S2**: Schematic illustration of the formulated dual ^68^Ga/Gd imaging probe~~.~~


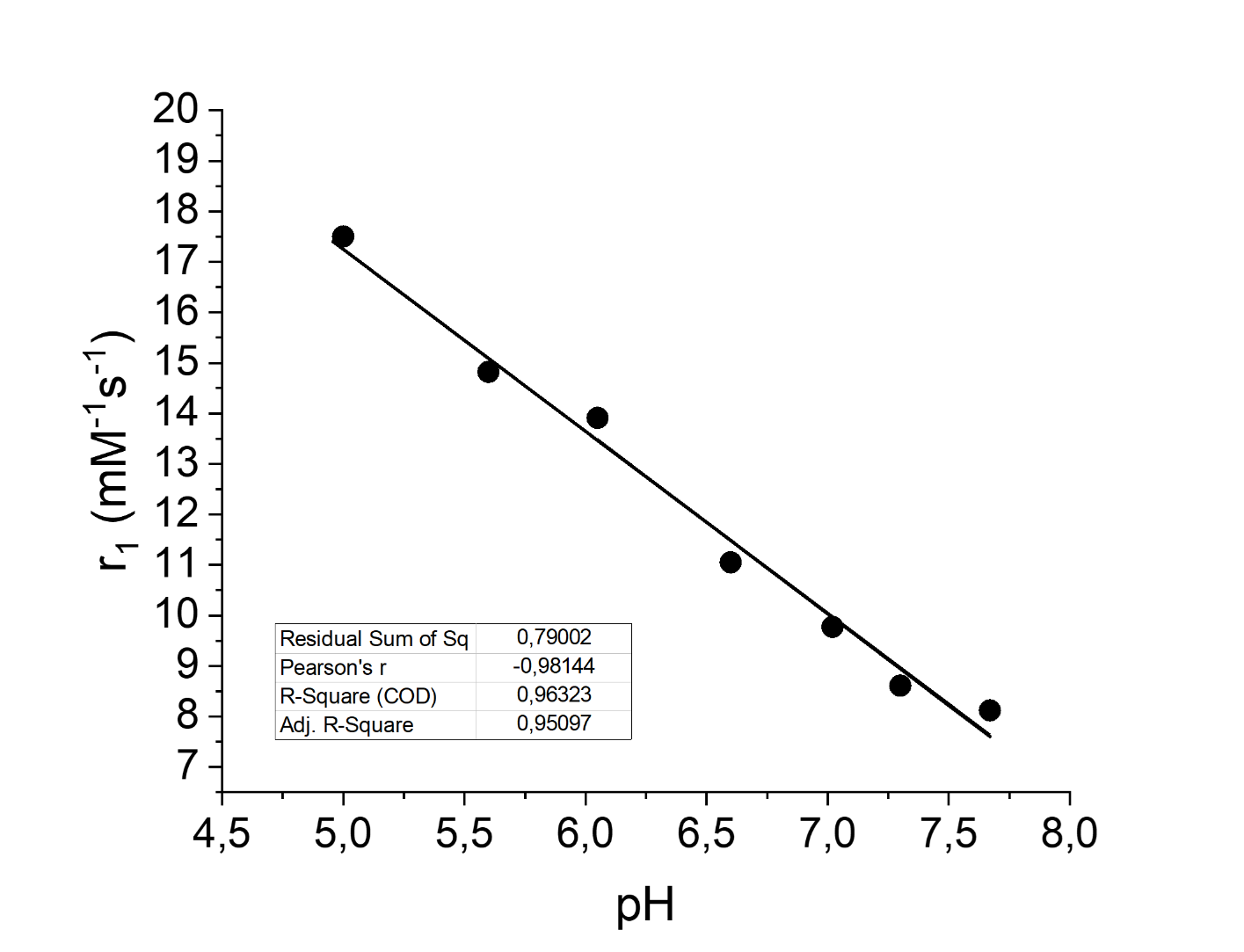


**Figure S1:** Calibration line of the relaxivity of Gd-DO3A-SA-Biot/Avidin adduct measured in NaCl 0.1 M at 1 T as a function of solution pH. The points were interpolated with the following linear correlation: r_1_ = -3.62(±0.4)×pH + 35.39(±2.84).


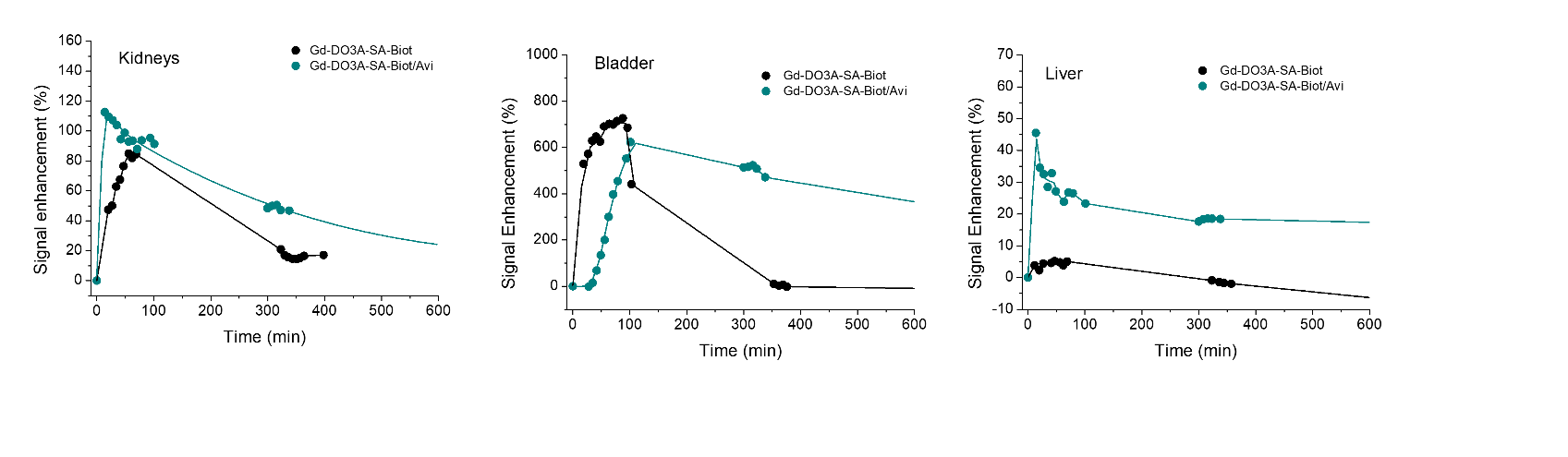


**Figure S2:** MR signal enhancement measured in the kidneys, bladders, and livers of mice injected with 0.05 mmol/Kg Gd-DO3A-SA-Biot or Gd-DO3A-SA-Biot/Avidin adduct.
